# Population structure of the hydrocoral *Millepora platyphylla* in habitats experiencing different flow regimes in Moorea, French Polynesia

**DOI:** 10.1371/journal.pone.0173513

**Published:** 2017-03-08

**Authors:** Caroline E. Dubé, Alexandre Mercière, Mark J. A. Vermeij, Serge Planes

**Affiliations:** 1 EPHE, PSL Research University, UPVD-CNRS, USR 3278 CRIOBE, Perpignan, France; 2 Laboratoire d’excellence “CORAIL”, EPHE, PSL Research University, UPVD-CNRS, USR 3278 CRIOBE, Papetoai, Moorea; 3 CARMABI Foundation, Piscaderabaai z/n, Willemstad, Curaçao; 4 Aquatic Microbiology, Institute for Biodiversity and Ecosystem Dynamics, University of Amsterdam, Science Park 700, Amsterdam, The Netherlands; US Geological Survey, UNITED STATES

## Abstract

While the fire coral *Millepora platyphylla* is an important component of Indo-Pacific reefs, where it thrives in a wide range of environments, the ecological and biological processes driving its distribution and population structure are not well understood. Here, we quantified this species’ population structure in five habitats with contrasting hydrodynamic regimes in Moorea, French Polynesia; two in the fore reef: mid and upper slopes, and three in the lagoon: back, fringing and patch reefs. A total of 3651 colonies of fire corals were mapped and measured over 45,000 m^2^ of surveyed reef. Due to the species’ sensitivity to fragmentation in response to strong water movement, hydrodynamic conditions (e.g. waves, pass and lagoonal circulation) corresponded to marked differences in colony size distributions, morphology and recruitment dynamics among habitats. The size structure varied among reef habitats with higher proportions of larger colonies in calm nearshore reefs (fringing and patch reefs), while populations were dominated by smaller colonies in the exposed fore reefs. The highest densities of fire corals were recorded in fore reef habitats (0.12–0.20 n.m^-2^) where the proportion of recruits and juveniles was higher at mid slope populations (49.3%) than on the upper slope near where waves break (29.0%). In the latter habitat, most colonies grew as vertical sheets on encrusting bases making them more vulnerable to colony fragmentation, whereas fire corals were encrusting or massive in all other habitats. The lowest densities of *M*. *platyphylla* occurred in lagoonal habitats (0.02–0.04 n.m^-2^) characterized by a combination of low water movement and other physical and biological stressors. This study reports the first evidence of population structure of fire corals in two common reef environments and illustrates the importance of water flow in driving population dynamic processes of these reef-building species.

## Introduction

Coral reefs exhibit a remarkable diversity of organisms that reside within highly variable environments resulting in strong spatial variability in species’ distribution patterns [1]. For scleractinian corals, spatial differences in temperature, light, water flow and water quality conditions can influence their distribution and population dynamics [2–5]. *Millepora* hydrocorals, also called fire corals, are an important component of reefs communities worldwide where they, similar to scleractinian corals, contribute to reef accretion and community dynamics [6,7]. Fire corals can colonize a wide range of reef environments through sexual reproduction [7,8] and colony fragmentation [7,9]. Fire corals have been reported to grow faster than scleractinian corals [7,10] and often grow into large colonies that preempt space and compete with scleractinian corals [11,12]. On the other hand *Millepora* species also contribute to the survival of corals during *Acanthaster* outbreaks as this corallivorous predator tends to avoid *Millepora* species [7,13].

Hydrodynamic forces in the form of water-displacement, velocity and acceleration have been recognized as a key factor in determining the shape and occurrence of many reef-building organisms [14–16]. In coral reef ecosystems, the magnitude of water flow is mostly related to the wave energy dispersal [5]. On barrier reefs, the amount of wave energy is highest on the reef crest, where waves break, and subsequently attenuates towards fore reef and lagoonal environments [5,17]. Inside lagoons, internal waves and flows drive circulation and water exchange with the surrounding ocean [18,19]. Such variation in hydrodynamic regimes, combined with other physical (e.g. light, nutrients and disturbances) and biological factors (e.g. colony size and shape), differently affect the performance of reef benthic organisms [20–23] resulting in corresponding changes in population structure and community composition.

Water flow can drive the spatial distribution in adult populations through the distribution and dilution of larval settlement cues [24] and dispersal of reproductive propagules [25,26]. Many studies have related the contribution of recruitment to colony size variation in scleractinian corals (e.g., [27,28]) and the size structure of a population often reflects other species specific responses to environmental conditions and disturbances as well [29–31]. The size-frequency distributions of fire coral populations could therefore provide insights on which biotic (e.g., recruitment of larvae and asexually produced fragments) and abiotic (e.g., wave energy) factors influence their population structure and dynamics.

Water flow also influences colony growth and morphology [32,33]. Under the increasing influence of hydrodynamics, delicate branching corals transform into growth forms able to withstand strong water movement such as compact, robust plating or thick branching morphologies [34,35]. Such inter- and intraspecific variation resulting in different coral morphologies affects not only their mechanical strength but also their ability to compete for space [36–38] and capture light and food [23,39]. Branching and plating scleractinian corals, such as *Pocillopora damicornis* and *Acropora hyacinthus*, often grow into large and delicate arborescent colonies in areas of relatively high water flow [23,39,40], but this growth strategy also renders them extremely vulnerable to breakage when large waves and storm events occur, often resulting in fragmentation with some mortality [41]. Asexual reproduction through colony fragmentation can be a successful reproductive strategy to sustain local population growth in some species of scleractinian corals [42,43]. Fire corals are also known for their extensive intra- and interspecific morphological variability across hydrodynamic gradients with consequences due to their vulnerability to wave-induced breakage [7,9,36,44]. Determining to what degree the population structure of fire corals depends on the differences in water flow among common reef habitats has so far not been determined.

In this study, we investigated whether and how different reef habitats with contrasting water regimes affect the population structure of *Millepora platyphylla*, Hemprich & Ehrenberg 1834, the only species of fire coral found in French Polynesia [45,46]. Surveys of *M*. *platyphylla* were conducted in five habitats on the north shore of Moorea (Society Archipelago, French Polynesia) with differing amounts of water flow: fore reef habitats with high water movement, especially on the upper slope and decreasing with depth to the mid slope. Lagoonal habitats (back reefs, fringing reefs and patch reefs) are sheltered from waves and oceanic swell, except during storms, and water movement in these habitats is less than on the fore reef [5,17]. We examined colony size distribution, morphological variability and recruitment dynamics to assess to what degree the variability in the population structure of *M*. *platyphylla* among reef habitats can be attributed to different flow regimes.

## Materials and methods

### Model species

*Millepora platyphylla* is a gonochoric broadcast spawner that reproduces sexually by producing medusoids and planula larvae [7]. The medusoids are released into the water column and the gametes are released in one hour post-spawning during the medusoid’s swim. Then, external fertilization and embryogenesis occur after which the larvae sink and move epibenthically (i.e. crawling) on the reef substratum and metamorphose into calcifying polyps within one day after spawning [8]. *M*. *platyphylla* can also reproduce asexually through fragmentation [7,9].

### Study sites and field surveys

**Fig 1:**
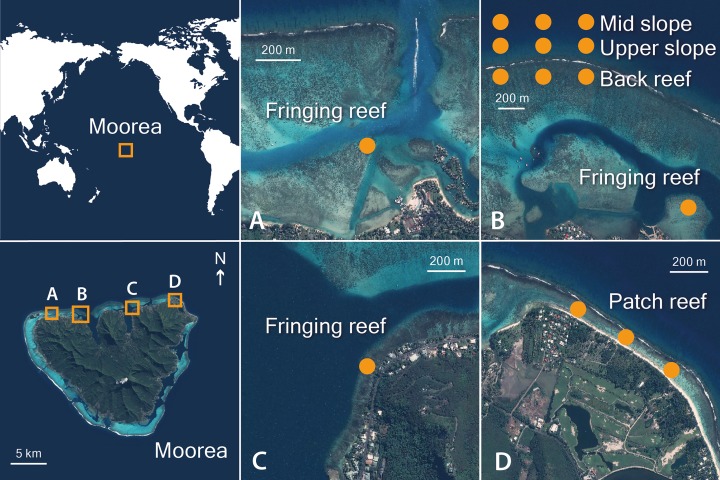
Aerial views of the locations of each transect in the five surveyed habitats in Moorea, French Polynesia. The names of these surveyed locations are: (A) Tiahura, (B) Papetoai, (C) Cook’s Bay and (D) Temae. Map data WorldView–2, Digital Globe.

Between April and December 2013, a series of surveys were conducted on the north shore of Moorea, French Polynesia, in the South Pacific Ocean (17,5267 S, 149,8348 W), at four different locations (Tiahura, Papetoai, Cook’s Bay and Temae). Five habitats with contrasting water flow regimes were selected; two in a fore reef environment: the mid slope (13 m depth) and the upper slope (6 m depth), and three in the lagoon (< 1 m depth): the back reef, fringing reef and patch reef (Fig 1 and S1 Table). These habitats greatly differ in terms of water flow. The fore reef experiences strong wave action from incoming waves that break on the reef crest with gradual swell wave attenuation towards deeper waters [5,47]. Because of this strong linear relationship between wave forcing and water depth [47], the colonies of *M*. *platyphylla* growing within fore reef habitats are exposed to lower wave energy on the mid slope compared to the those growing on the upper slope, near where the waves break. In the lagoon, the wave energy disperses from the reef crest towards nearshore reefs [5]. A recent study on wave energy across reef environments revealed that the reef crest dissipated 70% of the incident swell wave energy with gradual wave attenuation from the back reef to the shore [17]. Consequently, we assumed that wave energy is higher on the back reef, near the reef crest compared to the fringing reef, a nearshore reef. Although the patch reef is located in a nearshore narrow channel, the wave energy there is also higher than on the fringing reef due to its proximity to the reef crest and to the currents that run on either side of the channel (i.e. pass circulation). Variations in other physical constraints exist between the fore reef and lagoonal habitats in terms of e.g., temperature, water clarity, nutrient and disturbances [48,49], which make them highly contrasting reef environments.

Within each habitat, three 300 m long by 10 m wide belt transects were laid over the reef parallel to shore, at least 30 m apart, resulting in a total of 45,000 m^2^ reef area being surveyed. The north shore of Moorea is ~16 km long with a fore reef area of ~3.15 km^2^ and a back reef with a hard-bottom area of ~4.58 km^2^ [50]. We performed six belt transects of 0.003 km^2^ on the fore reef, which is ~0.1% of the total fore reef area on the north shore of Moorea. We also performed six belt transects of 0.003 km^2^ on the back reef area (i.e. both back and patch reef habitats), which is ~0.07% of the total back reef area. All colonies of *M*. *platyphylla* that were at least 50% within the transect borders were measured, photographed and georeferenced using SCUBA. No specific permit was required at the time of field work for sampling protocols described herein and our surveys did not involve endangered/protected species and did not require animal tissue/skeleton collection.

### Spatial distribution patterns

All *M*. *platyphylla* colonies were georeferenced by determining their position along the transect-line (0 to 300 m) and straight-line distance from both sides of the transect (0 to 10 m). From these measures, each colony was mapped with x and y coordinates, from which the distribution index (DI) and mean neighborhood distance (ND) were calculated using the spdep package [51] in R [52]. The DI is based on Ripley’s method [53] and calculated for each transect to determine whether colonies were having a contagious (DI > 1), random (DI ≈ 1) or homogenous (DI < 1) pattern of distribution [54]. The mean distance to each colony’s 10 nearest neighbors was estimated and the mean ND was calculated for each transect. The mean colony density (n.m^-2^) and cover (%) were also calculated for each transect (i.e. 3000 m^2^). Using these variables, variability in the spatial distribution among habitats was quantified by one-way PERMANOVA tests in PRIMER 6 software [55], since assumptions of parametric testing could not be met. Pair-wise tests followed the PERMANOVA to assess the degree of similarity among habitats. In order to determine how different habitats with contrasting water regimes affect the spatial distribution of *M*. *platyphylla*, we assumed that swell wave energy exposure decreases with habitat depth and its proximity to the coastline, as demonstrated in previous studies [17,47]. Consequently, the density, cover, DI and ND were regressed against the mean depth and mean distance from shore estimated from the three transects within each of the five surveyed habitats and Pearson’s *r* coefficient was used to test for significant correlations.

### Colony size distribution

The size-frequency distributions of *M*. *platyphylla* populations were generated from estimates of colony sizes computed from 2D photographs. Photographs were taken from above the colony and included a plate of known dimensions positioned next to each colony. For bigger colonies, pictures were taken from a larger distance, and for 5 colonies (out of the 3561) 2 photographs were required to photograph the whole colony. Each colony size, standardized as the projected surface, was then measured (in cm^2^) using ImageJ 1.4f software [56]. The size-frequency distribution for each transect was given as percentages of all colonies belonging to 10 size classes on a logarithmic scale. Data were then analyzed using basic statistical measures of size hierarchies [57]: the coefficient of variation (CV) and skewness (g_1_), indicative of the relative abundance of small and large colonies within a population. CV and g_1_ were computed for each habitat per transect together with standard descriptive statistics, such as 95% percentile of the mean (describes the maximum colony size reached within a population, see [58]) and the probability that the data are normally distributed (Kolmogorov-Smirnov test, Pnorm). Differences in size-frequency distributions among habitats were quantified using one-way PERMANOVA based on normalized abundances. Spearman’s rank coefficient and pair-wise tests followed the PERMANOVA to assess the degree of similarity among habitats.

### Recruitment dynamics

The mean abundance and proportion of recruits, juveniles and adults were estimated for each transect whereby the three life stages were defined based on colony size. Colonies with a total size (surface) below 1 cm^2^ were considered as recruits and were most likely the result of sexual reproduction. Larger colonies with a size between 1 and 20 cm^2^ were classified as juveniles based on previous studies on coral recruitment using both settlement plate experiments [59] and field surveys [60]. While the origin of each colony (sexual or asexual) could not be confirmed from field surveys, both size classes were considered as non-reproductive in contrast to colonies above 20 cm^2^ based on previous studies on other reef-building taxa [61]. Differences in abundances and proportions (i.e. the fraction in the entire population) of early life stages (i.e. both recruits and juveniles) among habitats were quantified using one-way PERMANOVA, followed by a pair-wise test. Pearson’s correlation coefficient was used to determine whether the abundance of early life stages increased with the abundance and cover of adults, and whether differences in their proportions among habitats correlate with water movement, i.e. with depth and distance from shore used as proxies.

### Colony morphology

**Fig 2:**
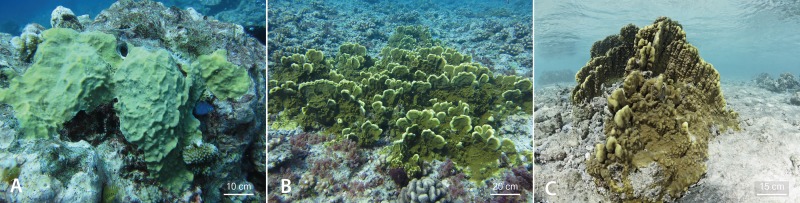
Morphologies of *Millepora platyphylla* colonies in habitats experiencing contrasting hydrodynamic regimes. (A) Encrusting wave-tolerant morphology in the mid slope, a fore reef habitat at 13 m; (B) sheet tree morphology vulnerable to wave-induced breakage in the upper slope, a fore reef habitat at 6 m and (C) massive wave-tolerant morphology in the patch reef, a lagoonal habitat (photo is courtesy of Gilles Siu).

**Fig 3:**
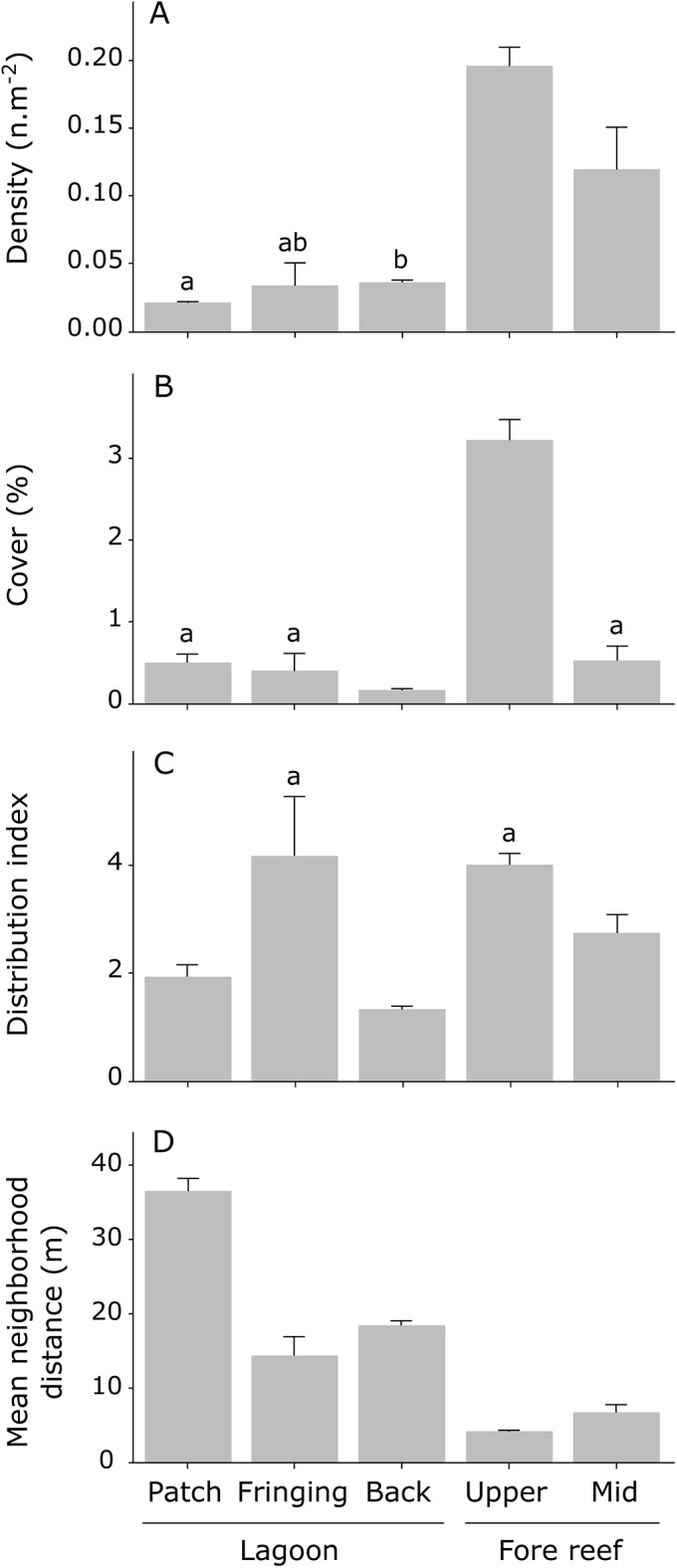
Index describing the spatial distribution of *Millepora platyphylla* colonies across the five surveyed habitats. (A) Density (B) cover (C) distribution index and (D) mean neighborhood distance. Values were average per habitat and error bars show the standard error for transect replicates. Similar letters indicate no statistical difference in post-hoc comparisons among habitats (*P >* 0.5).

For each colony’s morphology, the maximum height, from the base to the highest part of the colony (rounded to the nearest half cm), was recorded and linked to the colony size data previously described. Colonies below 20 cm^2^ were removed from this analysis to only retain the mean height and size of adults for each transect. Adult colonies were assigned to one of these three morphologies: 1) encrusting: thin colonies growing against the substratum (Fig 2A); 2) “sheet tree”: encrusting bases with platelike outgrowths facing wave energy (see [36]) (Fig 2B) and 3) massive: solid colonies, roughly hemispherical in shape (Fig 2C). Differences in proportions of each of the three morphologies among habitats were quantified with one-way PERMANOVA and pair-wise tests.

### Population structure assessment

Similarities in population structure based on the following parameters: density, cover, DI, ND, mean adult colony size and height, and proportion of recruits, juveniles and adults were calculated and visualized using a hierarchical complete-linkage agglomerative clustering (CLUSTER) method and a non-parametric multidimensional scaling (MDS) ordination on normalized data in PRIMER 6 software. Multivariate PERMANOVA on aforementioned characteristics was used to determine differences in population structure of *M*. *platyphylla* among the five surveyed habitats, i.e., those on the fore reef (mid and upper slopes) and those in lagoonal habitats (back, fringing and patch reefs).

## Results

### Spatial distribution of *Millepora platyphylla*

*M*. *platyphylla* was found in all habitats, but its population composition differed among habitats. A total of 3651 colonies of *M*. *platyphylla* were counted in the five surveyed habitats. Most colonies (48.2%) occurred on the upper slope, whereas *M*. *platyphylla* colonies on patch reefs accounted only for 5.2% of all colonies (S2 Table). Colony density differed among habitats (PERMANOVA test, *P* < 0.01) and was higher on the upper slope (0.20 ± 0.03 n.m^-2^, N = 1761) and mid slope (0.12 ± 0.05 n.m^-2^, N = 1075), i.e. fore reef habitats, compared to lagoonal habitats (back reef: 0.03 ± 0.01 n.m^-2^, N = 324, fringing reef: 0.04 ± 0.03 n.m^-2^, N = 302 and patch reef: 0.02 ± 0.00 n.m^-2^, N = 189) (Fig 3A). *M*. *platyphylla*’s cover also differed among habitats (PERMANOVA test, *P* < 0.01) and was again highest on the upper slope (3.2 ± 0.4%, Fig 3B). Colonies on the fringing reef, mid slope and upper slope occurred in a contagious pattern of distribution (DI: 2.74–4.18), while colonies in the back and patch reefs were more evenly distributed (≤ 1.93) (Fig 3C, PERMANOVA test, *P <* 0.05). Colonies occurred closer together on the mid slope (6.64 ± 1.86 m) and upper slope (4.09 ± 0.34 m) where the average distance among neighboring fire coral colonies was 4.3 times smaller compared to lagoonal habitats (back reef: 18.39 ± 1.10 m, fringing reef: 14.31 ± 4.41 m and patch reef: 36.51 ± 2.95 m) (Fig 3D, PERMANOVA test, *P* < 0.01).

### Size structure of *Millepora platyphylla*

**Fig 4:**
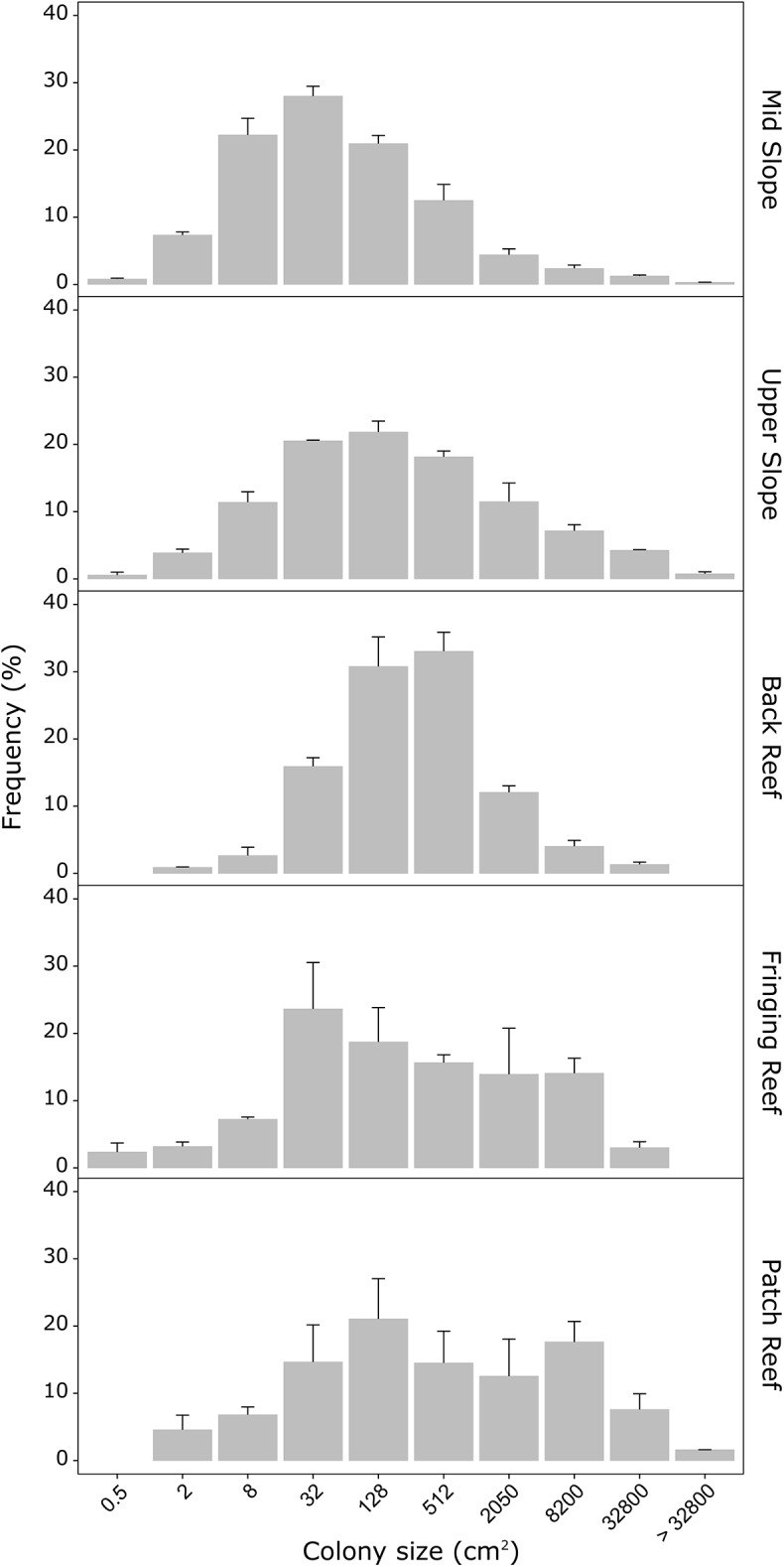
Size-frequency distributions of *Millepora platyphylla* across the five surveyed habitats. Colony size (cm^2^) data were distributed among 10 size classes based on a logarithm scale (log_2_). Frequencies (%) for each size class were averaged by habitats with total population size (N in S2 Table) and error bars show the standard error for transect replicates.

Across all habitats, 85% of the surveyed colonies were smaller than 1000 cm^2^ and approximately one third (30%) of aforementioned colonies fell in recruit and juvenile size classes, i.e. were smaller than 20 cm^2^. The size-frequency distributions of *M*. *platyphylla* populations differed among certain habitats, but were similar among the lagoonal fringing and patch reefs with populations dominated by a combination of small (≤ 32 cm^2^, comprising both small recruits and juveniles) and large colonies (≥ 2050 cm^2^) resulting in bimodal size-frequency distributions (Fig 4, Spearman’ rank coefficient 87.9%, *P* < 0.05). All fire coral populations were characterized by relatively symmetrical size distributions (g_1_: –0.01–0.71), but the degree of skewness was again lower on the fringing and patch reefs (Table 1). The maximum colony size differed among habitats and was smallest on the mid slope (95%: 1295 cm^2^) and back reef (2512 cm^2^) compared to other populations (upper slope: 8514 cm^2^, fringing reef: 7107 cm^2^ and patch reef: 9890 cm^2^) (Table 1). With 64% of all colonies falling in a few medium size classes (32–512 cm^2^, Fig 4), colonies comprising back reef populations were very similar relative to each other as indicated by the lowest coefficient of variation (CV: 0.33, Table 1) of all habitats. Overall, the composition of *M*. *platyphylla* populations in terms of colony density and size differed among the five habitats, except between the two lagoonal habitats, the fringing and patch reefs, located closest to shore.

**Table 1:** Index describing the population size structure and recruitment for *Millepora platyphylla* across the five habitats surveyed.

Habitat	Colony size (cm^2^)					Recruitment			
	Non-transformed		ln-transformed			Abundance (n)		Proportion (%)	
	Mean (SE)	95%	Pnorm	CV	g_1_	Recruit	Juvenile	Recruit	Juvenile
Patch	3090.48 (1293.52)	9889.89	<0.01	0.51	–0.01	1.00 (1.73)	11.30 (3.06)	1.47 (2.55)	17.93 (4.52)
Fringing	1590.07 (328.94)	7107.16	<0.01	0.49	0.14	1.33 (0.58)	21.33 (17.21)	1.74 (1.86)	24.00 (8.20)
Back	509.87 (97.07)	2512.02	<0.01	0.33	0.68	---	13.00 (7.55)	---	11.77 (5.94)
Upper	2308.20 (115.84)	8513.79	<0.01	0.54	0.42	9.33 (3.51)	161.00 (32.70)	1.57 (0.53)	27.26 (3.53)
Mid	819.04 (73.30)	1295.23	<0.01	0.63	0.64	12.00 (4.36)	159.00 (53.33)	3.44 (0.40)	45.86 (5.15)

Mean, estimated from adult colonies (> 20 cm^2^); 95%, maximum size; Pnorm, Probability that the data are normally distributed; CV, coefficient of variation; g_1_, skewness; Recruit, < 1 cm^2^; Juvenile, 1–20 cm^2^. Values were average per habitat and ± SE for variation among transects.

### Recruitment of *Millepora platyphylla*

**Fig 5:**
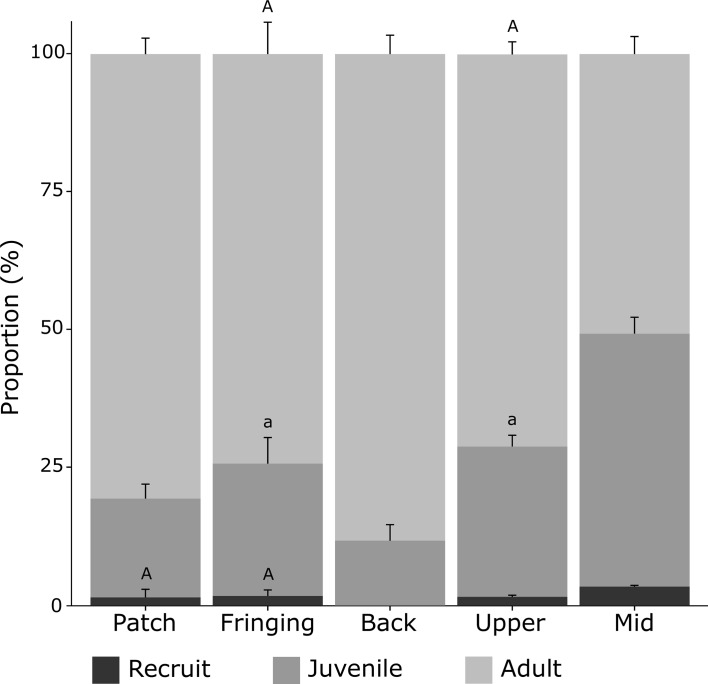
Recruitment dynamics across the five surveyed habitats. Proportions of recruits (< 1 cm^2^), juveniles (1–20 cm^2^) and adults (> 20 cm^2^) were averaged by habitats with total population size (N in S2 Table) and error bars show the standard error for transect replicates. Similar letters over each set of bars indicate no statistical difference in post-hoc comparisons for a given life history stage among habitats (*P >* 0.05).

**Fig 6:**
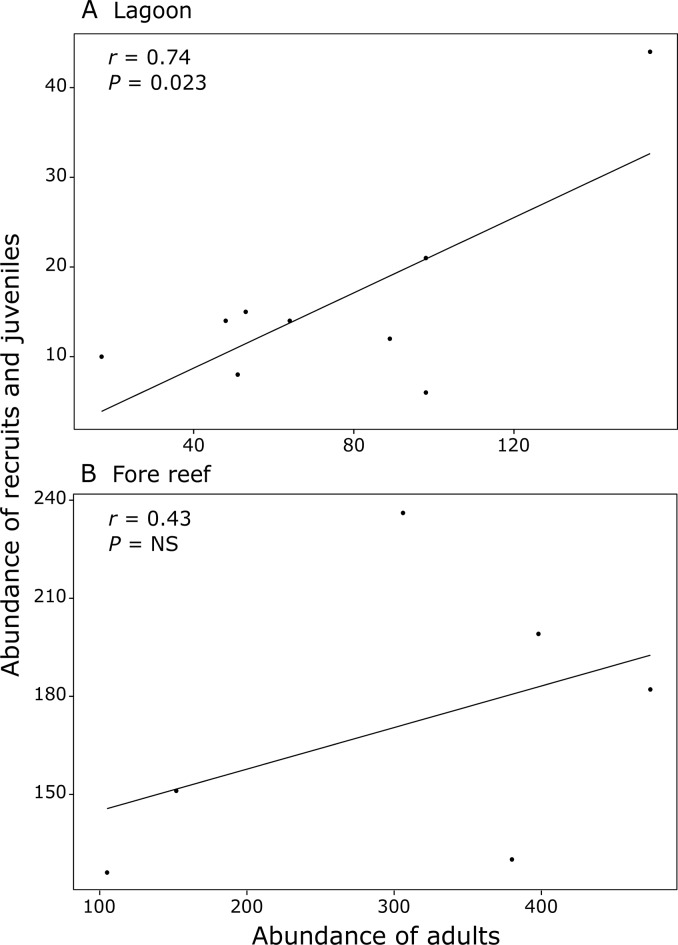
Stock-recruitment relationship between the abundance of adults and coral new recruits and juveniles. (A) Significant positive relationship in the lagoon (i.e. back, fringing and patch reefs) and (B) no stock-recruitment relationship in the fore reef (i.e. mid and upper slopes). Each dot represents the mean abundance for each transect surveyed. Note the different scales on x and y axes.

In total, 71 recruits (2%) and 1094 juveniles (30%) were observed within the five surveyed habitats (S2 Table). The abundance of recruits and juveniles differed among habitats (PERMANOVA test, *P* < 0.05 and *P* < 0.01, respectively) with 96% of all recruits and juveniles occurring in fore reef habitats (48% for both the mid and upper slopes) and only small numbers were observed in lagoonal habitats (Table 1). The fraction of the entire population consisting of recruits and juveniles differed among habitats (PERMANOVA test, *P* < 0.01). Recruits were found in low proportions in most habitats with the highest value recorded on the mid slope (3.4 ± 0.4%), while no recruit was observed on the back reef (Table 1; Fig 5). The mid slope habitat sheltered the highest proportion of juvenile colonies (45.9 ± 5.1%), while lower values were found in all other reef habitats (11.8–27.3%) with no significant difference between the fringing reef (24.0 ± 0.2%) and upper slope (27.3 ± 3.5%, Table 1; Fig 5). Only in lagoonal habitats did the abundance of adults, not their total cover, and the abundance of both recruits and juveniles increase simultaneously suggesting the presence of a stock-recruitment relationship (Fig 6A), that was not observed in fore reef habitats (Fig 6B). No significant stock recruitment relationship was found in both lagoonal and fore reef habitats when only considering recruit and adult’s abundances.

### Morphology of *Millepora platyphylla*

**Fig 7:**
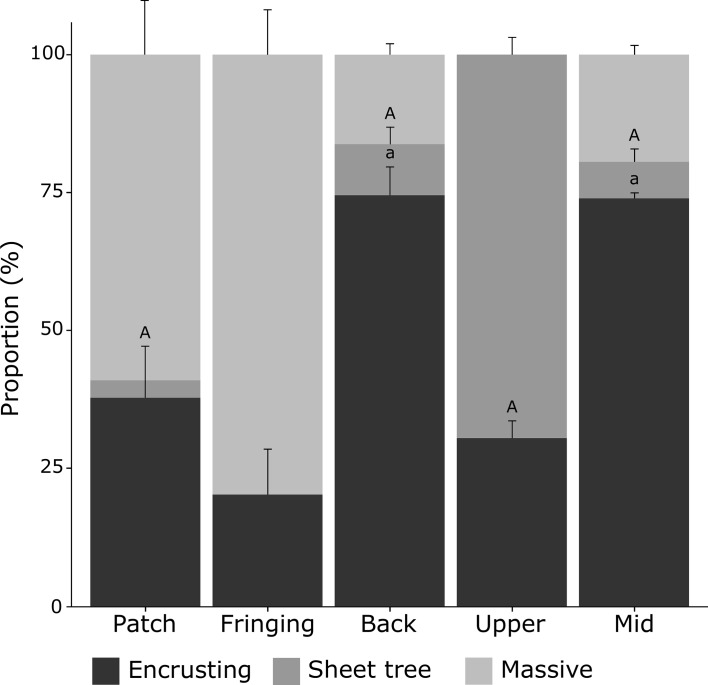
Morphology of *Millepora platyphylla* adult colonies across the five surveyed habitats. Proportions of colonies with encrusting, sheet tree and massive morphology were averaged by habitats and error bars show the standard error for transect replicates. Similar letters over each set of bars indicate no statistical difference in post-hoc comparisons for a given morphology among habitats (*P >* 0.05). See Fig 2 for photos of each of the morphologies.

*M*. *platyphylla* colonies ranged in size from 0.18 cm^2^ to 189 062 cm^2^ (projected surface) and 0.1 cm up to 130 cm in height. Mean colony size and height of adults (i.e., all colonies > 20cm^2^) differed among habitats (PERMANOVA tests, *P* < 0.01). Fire corals were approximately 4 times larger on average in the upper reef slope (2308 ± 115 cm^2^), fringing (1590 ± 329 cm^2^) and patch reef (3090 ± 1294 cm^2^), compared to colonies growing in the back reef and mid slope (510 ± 97 cm^2^ and 819 ± 73 cm^2^, respectively, Table 1). The average height of fire coral colonies was highest in fringing (24 ± 5 cm) and patch reefs (25 ± 8 cm), i.e. nearshore habitats (S2 Table). Morphologies of adult colonies differed among habitats (PERMANOVA test, *P* < 0.01). Massive morphologies dominated nearshore reefs (fringing reef: 79.7 ± 8.3% and patch reef: 59.0 ± 9.9%) whereas colonies were mostly encrusting on the mid slope (79.9 ± 1.1%) and back reef (74.5 ± 5.2%) (Fig 7 and S3 Table). On the fringing reef, no colony with the sheet tree morphology was observed. On the upper slope, 69.5% (± 3.2) of the colonies displayed the sheet tree morphology, while the remaining colonies were only encrusting.

### Population structure assessment

**Fig 8:**
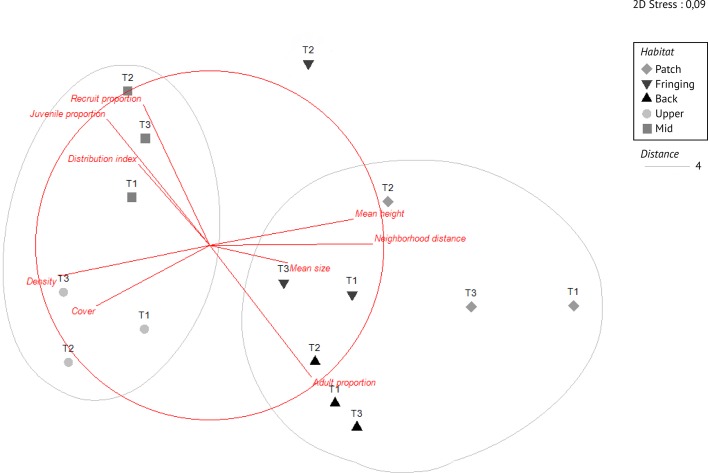
Non-metric multidimensional scaling (MDS) plot of *Millepora platyphylla* population structure across the five surveyed habitats. Different shapes indicate the three transects for each habitat and grey lines show clusters given by dendogram based on Eucledian distance of 4 at a stress level of 0.09. The surimposed red lines define the Eucledian distance coefficient on normalized data based on Spearman ranking, with each vector having lengths ≥ 0.4: density, cover, distribution index, mean neighborhood distance, mean height and size of adults, and proportion of recruits (< 1 cm^2^), juveniles (1–20 cm^2^) and adults (> 20 cm^2^). The second transect of the fringing reef is shown as a single group mostly related to a small population size (i.e. 27 colonies, S2 Table).

Combining all variables into a single multivariate analysis, the population structure of *M*. *platyphylla* varied significantly among reef habitats (PERMAVOVAs, *P* < 0.01). Based on MDS, two main clusters can be distinguished: one with populations from wave exposed fore reef habitats, i.e. mid and upper slopes, and a second cluster consisting of populations from lagoonal habitats, i.e. back, fringing and patch reefs (Fig 8). The main differences between these two clusters are that fore reef populations are characterized by a high relative abundance of recruits and juveniles (mid slope), or a higher density and cover (upper slope). Populations from lagoonal habitats are characterized by large colony size and height (both fringing and patch reefs) and widely spaced colonies. Back reef populations are characterized by the dominance of adult colonies.

Using all variables of each transect surveyed within the fore reef habitat (i.e. six replicates), we found that adult colonies became smaller (i.e. colony size decreases) with increasing depth (r = –1.00, *P* < 0.001; N = 6). Fractions of recruits and juveniles increased with increasing depth (r = 0.91, *P* < 0.05 and r = –0.92, *P* < 0.05; N = 6), while total cover decreased (r = –0.97, *P* < 0.01; N = 6). Colonies grew in an encrusting morphology at mid depth and in the sheet tree morphology in shallow waters (Fig 7). Among shallow lagoonal habitats, we found that adult colonies became smaller towards the back reef, far from shore (r = –0.76, *P* < 0.01; N = 9), where wave energy was higher and colonies mostly occurred in the encrusting morphology (Fig 7). Total cover increased with increasing distance from the coast (r = –0.69, *P* < 0.05; N = 9).

## Discussion

### Distribution patterns: Fore reef versus lagoonal habitats

In Moorea, *M*. *platyphylla* colonized a wide range of habitats reflecting its ability to adapt and survive in a large variety of environmental settings. This study is, to our knowledge, the most extensive sampling ever conducted to assess local patterns in population structure of Milleporid corals. Reef habitats where *M*. *platyphylla* colonies were found were selected because of their difference in water regimes according to their depth and proximity to the coastline (see Materials and Methods for details). Due to *M*. *platyphylla*’s sensitivity, especially of larger colonies to fragmentation induced by wave action and/or water movement (i.e. currents) (Fig 2), we sought for possible relationships between hydrodynamic conditions and the population structure of fire corals on Moorea. Differences in population size structure, recruitment and morphology existed among habitats and confirmed expected relationships between such characteristics and the amount of water flow in several of the five surveyed habitats (i.e. mid slope, upper slope, back reef, fringing reef, and patch reef). The highest densities of fire corals, including that of recruits and juveniles, occurred on the exposed fore reef (i.e. mid and upper slopes) whereby colonies were often observed growing in contagious pattern of distribution. In calm lagoonal environments (i.e. back, fringing and patch reefs) fire coral colonies occurred in low densities, where the number of recruits and juvenile was low and colonies grew in a random pattern of distribution. Variability in density among fore reef and lagoonal habitats has been described for numerous other sessile organisms and related to a large number of environmental factors such as water flow, solar irradiance, sedimentation and/or species’ life history traits (e.g., reproductive mode, competitive ability, morphological plasticity; [20,62–65].

### Size structure and morphological variations

Although differences in size-frequency distributions among habitats were found, e.g. few larger colonies in the calm fringing and patch reefs, smaller colonies in the mid slope and medium size colonies in the back reef, the degree of skweness was similar among all habitats with populations consisting of both small and large colonies. These distributions likely reflect low mortality in small size classes, as well as the persistence of the larger ones [66]. Our results showed that the proportion of recruits and juveniles was highest on the mid slope, an exposed reef where wave energy is reduced due to increased depth [47]. Earlier reports have also shown the influence of depth and water flow on the recruitment dynamics in some scleractinian coral species in many reef locations [67–69]. These studies revealed an increase in the occurrence of recruits and juveniles with increasing depth. Another study compiling juvenile data of all coral species surveyed in Palmyra Atoll (Central Pacific) has shown that most juveniles were growing at middle depth (i.e. 14 m) in a fore reef habitat [63], as for *M*. *platyphylla*. Water flow is also considered as an important factor influencing a colony’s morphology [70,71], generally showing a transition from easily fragmented morphologies towards more robust morphologies with increasing water movement [34,35]. This study shows a similar trend whereby large and high colonies were more common in protected nearshore habitats (i.e. fringing and patch reef) and small and encrusting in exposed mid slope and back reef habitats. On the upper slope, near where the waves break, fire corals are large, but largely encrusting, and of the unusual sheet tree morphology of *Millepora* that was only observed in low proportions in all other habitats (0–9%).

### Population structure and dynamics

Fire corals, like many other reef-building organisms, reproduce through both asexual and sexual reproduction with a dimorphic life cycle, with a pelagic dispersive phase (i.e. medusoids and larvae), followed by a sessile adult phase [7,8]. If dispersal distances are small due to low water movement or retention, the spatial distribution of adults could influence the distribution of young colonies as previously shown for scleractinian corals [68,69,72,73]. On Moorea the abundance of *M*. *platyphylla* recruits and juveniles could not be related to adult population size in fore reef habitats. The proportions of recruits and juveniles were highest at mid-depths (13 m) on the fore reef where wave energy and solar irradiance are lower compared to the shallower depth habitats (as described in [63]). Low wave energy can indeed increase settlement success of both coral larvae and fragments [74]. At shallow depths (6 m) on the fore reef, high wave energy and irradiance can reduce the abundance of settlement cues [74], but also indirectly affect settler survival through high grazing pressure by herbivorous fishes at this depth which constitutes a major source of mortality for juvenile corals on the upper slope in Moorea [59]. The abundance of coral fragments that re-attached to the reefs can also be reduced due to high wave energy and subsequent increased mortality [75]. Such physical and biological constraints in a dynamic environment likely reduce local recruitment rates and prevent high coral cover [76]. However, the highest cover of *M*. *platyphylla* (3.2%) occurred on the upper slope where wave breaking first occurs, i.e. wave energy is the highest. Many studies investigating spatial distributions in coral reef communities often find that high energy reef zones restrict species’ distributions and cover [77,78]. *M*. *platyphylla* shows the opposite trend: we observed high density and cover in the upper slope, a high energy reef zone, where colonies are growing in a contagious pattern of distribution. Such differences in fire coral distribution patterns in habitats of high energy are mostly related to the wave-vulnerable sheet tree morphology of *Millepora*. This growth form occurred nearly exclusive on the upper slope, while colonies were massive or encrusting in other habitats. The unusual sheet tree morphology observed in the upper slope has been described as a successful strategy exploited by *Millepora* to preempt the space and to compete with other coral taxa [12,36]. Waves can easily break the blades and enhance population growth through clonal propagation [9], while the encrusting bases remain intact and grow through horizontal stolonal spreading [12]. The fact that *M*. *platyphylla* can rapidly overtake newly available space through clonal propagation and stolonal spreading may explain the increase of fire coral cover on Moorea’s reefs following the massive decline in coral cover from the *Acanthaster* outbreaks and cyclone *Oli* in 2010 [79]. Between 2006 and 2010, *M*. *platyphylla*’s cover was stable at approximately 1% at 6 m on the fore reef, i.e. more than 3 times lower than in 2013 at the same location. On the other hand, fragmentation usually induces corals to regress in size and increases mortality, especially in small size classes [80]. Here, the sheet tree morphology is more easily fragmented, but the unilateral growth of *Millepora* allows them to reach larger colony sizes. This study shows that asexual reproduction through fragmentation and stolonal spreading likely plays a key role in structuring *M*. *platyphylla* populations where water flow is high and where fire corals face wave-induced breakage.

In the lagoonal environment, the wave energy is reduced by the reef crest [17] likely explaining the positive stock-recruitment relationship found in these habitats. There is evidence showing that the fecundity in populations of sessile marine broadcast spawners, such as *Millepora* species, is strongly determined by the local density of adults [81,82], and especially where water movement is reduced and local retention occurs. The low abundance of early life stages observed in all lagoonal habitats may result from competition with macroalgae and sediment smothering affecting back, patch and fringing reefs inside the lagoon of Moorea [83] and/or solar irradiance [74,76]. The presence of macroalgae and high sedimentation can additionally reduce adults’ fecundity [84–86], larval settlement cues [87], larval survival [88] and settlement space [89]. Poorer water quality compared to fore reef habitats also likely contributes to the low abundance of Milleporid corals inside the lagoon [90,91]. The back and patch reefs are the nearest to the reef crest where waves break resulting in low residence times and high flushing rates from large incoming waves that break on the north shore of Moorea during the austral summer [18]. These dynamics of water flow are known to negatively affect local recruitment rates of sexual propagules in scleractinian corals on the back reef of Moorea [25] and could also apply to *M*. *platyphylla*. In the lagoon, fire corals are characterized by wave-tolerant morphologies (i.e. encrusting and massive) suggesting that asexual reproduction through colony fragmentation is less likely of structuring importance compared to fore reef habitats. Colonies on the fringing reef, where wave energy is typically low, were distributed in patches. In Moorea, the fringing reef is exposed to large waves in the austral summer [18], which has the potential to enhance the breakage of the colonies during short periods. Subsequent calm periods can facilitate fragment survival and reattachment resulting in the patches of *M*. *platyphylla* observed.

### Implications for population maintenance and recovery

It must be noted that abundance of recruits and juveniles was likely underestimated in this study as they are difficult to find due to their small size during field surveys. Still, we identified 32% of colonies below 20 cm^2^ (recruits and juveniles), a higher fraction than observed for 14 different genera of scleractinians corals in Moorea (13–29%, see [68]). The abundance of fire corals around Moorea is also higher compared to more diverse and healthy reefs, such as the Great Barrier Reef [48] and the shallow fringing reefs in the US Virgin Islands [92]. Our results thus suggest that fire coral populations are relatively resilient in the face of recent and major disturbances that have impacted Moorea’s reefs (*Acanthaster* outbreaks, cyclones and bleaching events). The maintenance and recovery of fire coral populations are foremost sustained by the growth of remnant colonies, local recruitment through sexual reproduction where wave energy is low and clonal propagation in high wave energy zones.

## Supporting information

S1 Table Locations of each transect surveyed in the five habitats. (XLSX)

S2 Table Index describing the spatial distribution, recruitment and morphology for *Millepora platyphylla* across the five surveyed habitats. (XLSX)

S3 Table Average percentages of adult colonies for *Millepora platyphylla* with encrusting, sheet tree and massive morphology across surveyed habitats. (XLSX)
